# West European Hedgehog ( *Erinaceus europaeus* ) Decline in Great Britain: Evidence From Multiple National‐Scale Surveys

**DOI:** 10.1002/ece3.74321

**Published:** 2026-10-08

**Authors:** David E. Wembridge, Steve D. Langton, Henrietta Pringle, Nicholas J. Aebischer, Julie A. Ewald, David G. Noble, Silviu O. Petrovan

**Affiliations:** ^1^ People's Trust for Endangered Species London UK; ^2^ Hallgarth Leavening UK; ^3^ Game & Wildlife Conservation Trust Fordingbridge Hampshire UK; ^4^ British Trust for Ornithology Thetford UK; ^5^ Conservation Science Group, Department of Zoology University of Cambridge Cambridge UK

**Keywords:** citizen science, farmland biodiversity, long‐term trends, mammal monitoring, species conservation

## Abstract

There are increasing concerns about the population trends and conservation status of West European hedgehogs across their range. However, survey data for this species come from multiple independent projects with different aims and constraints; as such, data remain difficult to integrate and interpret. We collate and analyse hedgehog data from five separate recording schemes across Britain, representing the most complete information on population trends of the species to date. Analyses of three surveys, the longest going back to 1981, all show declines at rural sites, with changes in hedgehog abundance of −70.7% and −54.4% between 1981 and 2023, and 2002 and 2013, respectively, and −80.4% in occurrence between 2002 and 2023. Worryingly, the estimated average annual rate of decline accelerated from −2.88% between 1981 and 2023 to −6.81% between 2013 and 2023. Abundance changes in predominantly urban areas showed moderate increases in two surveys (6.7% between 2009 and 2023, and 20.0% between 2004 and 2024 respectively), while the same two surveys showed different trends in occurrence, declining until 2012/13 and thereafter either remaining below the baseline or recovering to around the baseline value. Possible reasons for this discrepancy are discussed. Overall, the results suggest significant changes in the hedgehog population of Great Britain between 1981 and 2024, indicating substantial, widespread declines in rural areas, with implications for future conservation efforts targeted at this species.

## Graphical abstract

We combined and assessed multiple national‐scale monitoring datasets for the West European hedgehog. They suggest a long‐term and concerning abundance decline in agricultural landscapes but apparently stable trends in urban areas. These results have important implications for wider farmland biodiversity in Britain, as well as hedgehog conservation strategies.

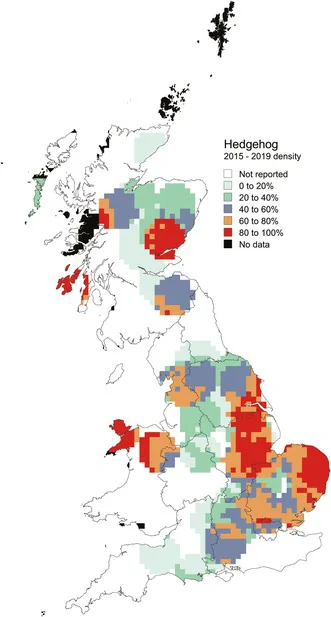

## 1 Introduction

The West European Hedgehog ( *Erinaceus europaeus* ) is a widespread, generalist species whose distribution extends through much of western to central Europe. However, based on evidence of population declines approaching or exceeding IUCN (International Union for Conservation of Nature) Red List criteria across much of its range, its conservation status was reassessed from Least Concern to Near Threatened, globally (Gazzard and Rasmussen 2024), and as Vulnerable in Great Britain (Mathews and Harrower 2020). Abundance declines and status deterioration of common species, such as this, have long been discussed for farmland bird species and butterflies in Europe and raise significant concerns about the ongoing impacts of agricultural intensification on biodiversity (Donald et al. 2001; Warren et al. 2021; Rigal et al. 2023), but remain rarely reported for mammal species. The apparent reasons for the abundance declines of the West European hedgehog (hereafter ‘hedgehog’) likely include lack of invertebrate prey, road mortality, toxin accumulation and others but there is significant uncertainty in understanding the specific level of impacts from different threats on different populations, partly due to inconsistencies in population monitoring for this species (IUCN/CPSG 2024).

Although hedgehogs are better studied than most wild mammal species in the UK (Gazzard et al. 2025), nocturnality, wide distribution and often low population densities pose significant challenges for their monitoring. Consequently, hedgehog abundance data remain sparse (Gazzard et al. 2025) and risk assessments highlight the need for increased monitoring in regions lacking abundance and trend data (Mathews and Harrower 2020). The absence of such abundance data for hedgehogs at regional and national scales has implications in terms of the ability to collate long‐term population trends, for understanding habitat‐specific densities, and for targeting conservation actions for this species in the areas where most needed (IUCN/CPSG 2024). They are, however, a highly charismatic species (e.g., RSB 2016) and frequently present in urban and exurban environments, making their monitoring amenable to citizen science approaches (e.g., Toms and Newson 2006; Gazzard 2022). Several ongoing, volunteer‐based citizen science projects collect records of hedgehogs in the UK, but for the most part these schemes are not specifically intended to monitor hedgehogs. Roos et al. (2012) assessed the potential of several such schemes to do so and found statistically significant declines in five estimates of either hedgehog population occupancy or abundance (from four monitoring schemes) over periods of four to 14 years between 1996 and 2010, depending on the particular recording scheme; two schemes showed nonsignificant declines. Where linear trends could be fitted, the average change over 10 years was estimated to be −40%. Based on this and a second published estimate of population change (Mathews et al. 2018), Mathews and Harrower (2020) took a value of 39% over three generations (13.2 years) as a reasonable inference of the decline in the hedgehog population in Britain up to that point.

Successive *State of Britain's Hedgehogs* reports, produced by People's Trust for Endangered Species (PTES) and the British Hedgehog Preservation Society (BHPS), have described population trend estimates and conservation efforts for hedgehogs, most recently with data up to 2019 (Wembridge et al. 2022). Here, we bring together all available data from five monitoring schemes and update trend estimates from the last of these reports, with the aim of producing a comprehensive evidence base of current and historic changes in the hedgehog population in England, Scotland and Wales at national and regional scales. We discuss the implications and limitations of these analyses for understanding the conservation status of the species. Ultimately, the critical appraisal of these results should inform future priorities for the targeted monitoring and conservation of hedgehogs in Britain.

## 2 Materials and Methods

### 2.1 Monitoring Schemes

Data from five recording schemes are analysed and presented here, covering periods between 1981 and 2024 but with differences between schemes (Table 1). Survey methodologies and geographical coverage, except for the *National Gamebag Census*, are described in Roos et al. (2012) and summarised here, including details of data sources (Table 1).

**TABLE 1:** Summary of data sources.

Survey	Organisation	Data inclusion period	Calendar period of data	Mean number of sites contributing data/year (range)	Mean number of sites recording hedgehogs/year (range)	Data type	Sampling characteristics
Garden BirdWatch	BTO	2007–2023	Weeks 20–35 (approx. mid‐May to late August)	2231 (816–3595)	1142 (444–2139)	Presence‐absence (from sightings and/or signs); maximum counts	Self‐selected sites; effort not recorded
Living with Mammals	PTES	2003–2024	April–June	348 (64–567)	147 (39–256)	Presence‐absence (from sightings and/or signs); maximum counts	Self‐selected sites; effort recorded
Breeding Bird Survey	BTO/JNCC/RSPB	2000–2023	April–June	2741 (1749–3438)	103 (28–276)	Presence‐absence (from sightings and/or signs)	Randomised sites; effort recorded
Mammals on Roads	PTES	2001–2013	July–September	399 (129–749)	242 (50–548)	Count	Self‐selected sites; effort recorded
National Gamebag Census	GWCT	1981–2023	All year	747 (454–904)	83 (10–174)	Count	Self‐selected sites; effort not recorded

Abbreviations: BTO, British Trust for Ornithology; GWCT, Game and Wildlife Conservation Trust; JNCC, Joint Nature Conservation Committee; PTES, People's Trust for Endangered Species; RSPB, Royal Society for the Protection of Birds.

#### 2.1.1 Garden BirdWatch

British Trust for Ornithology (BTO)'s *Garden BirdWatch* (GBW) is a large citizen science initiative that records weekly sightings of birds in domestic gardens throughout the UK; mammal recording is optional and is not necessarily carried out in the same weeks that birds are recorded. For the current analysis, only weeks in which a mammal species was recorded were included, with the assumption that participants who recorded another mammal species would also record hedgehogs if detected. Mammals are recorded either as a weekly maximum count (the largest number of individuals observed simultaneously) or as ‘presence’ in a particular week (from sightings or field signs). In total, 5350 sites in England, Scotland and Wales submitted data between 2007 and 2023, producing 35,016 site‐year combinations that were included in the trend analysis.

#### 2.1.2 Living With Mammals

PTES' *Living with Mammals* (LwM) project collects weekly records of mammals at sites in the built environment submitted by the public. Truncated maximum weekly counts (recorded as ‘1’, ‘2’ or ‘3+’ individuals) and/or signs of hedgehogs (e.g., droppings) were recorded during a 13‐week period from the end of March to the beginning of July (exact dates depending on the year). Sites were green spaces (excluding nature reserves) in England, Scotland and Wales that were within 200 m of buildings or enclosed by built areas (e.g., urban parks). In total, 1315 sites surveyed for at least 4 weeks in two or more years between 2003 and 2024, were included in the analysis; 71.6% of sites submitted data in more than 2 years; 47.1%, in five or more years; and 19.2%, in 10 or more years. Domestic gardens made up 83.4% of sites, while other site types included recreational spaces (parks, playing fields and golf courses; collectively, 4.11%), common ground (2.74%) and allotments (1.67%). A large majority of sites (93%) were in England.

Participants recorded weekly effort (length of observation at each of four periods, ‘dawn’, ‘day’, ‘dusk’ and ‘night’), as well as site characteristics (e.g., site type, size, composition, etc.) and presence of particular features (e.g., a pond, compost heap, bird feeder, etc.).

#### 2.1.3 Breeding Bird Survey

The *Breeding Bird Survey* (BBS) is an annual transect survey run by BTO, the Joint Nature Conservation Committee (JNCC) and the Royal Society for the Protection of Birds (RSPB). Two 1‐km transects, 500 m apart, are walked during the period April to June by volunteers in randomly selected Ordnance Survey (OS) grid cells in the UK (Massimino et al. 2025). Mammal recording was introduced in 1995 and is optional; from 2002, presence records were identified as: sightings of live animals; field signs; dead animals; or local knowledge of presence during the current season. Hedgehog records were most commonly assigned to the last of these. For the current analysis, only site‐year combinations that recorded at least one mammal or that indicated that the observer had opted in to mammal recording were used.

Roos et al. (2012) estimated an overall detection rate for hedgehogs in this survey (for data up to 2009) of 8.6%.

#### 2.1.4 Mammals on Roads

PTES' *Mammals on Roads* (MoR) project collected records of mammal road casualties submitted by volunteers on self‐reported road journeys, together with route information, on road journeys in July, August and September, in Great Britain. Records were collected either on paper forms or, from 2011 onwards, web‐based forms or using an application on an assisted GPS device. Route information was recorded differently in each case: paper forms captured journey start and end points, and waypoints at approximately 15‐km intervals, identifying the nearest settlement, mileage and road number (if an A or B road) for each location. Routes were identified on a map in the web‐based form or, in the case of application‐based records, recorded by the mobile device.

Although the survey continued until 2024, with records submitted exclusively via the application from 2018, overall participation and record numbers were comparatively small after 2013.

#### 2.1.5 National Gamebag Census

Game and Wildlife Conservation Trust (GWCT)'s *National Gamebag Census* (NGC) collects records of mammal species killed by shooting or gamekeeping activities (e.g., trapping) permitted by legislation on shooting estates (Aebischer et al. 2011). Volunteers submit annual numbers of animals killed for each species, including hedgehogs that are accidentally killed in tunnel traps set for small mammalian predators of gamebirds (e.g., stoat, *Mustela erminea* or brown rat *Rattus norvegicus* ). While the level of gamekeeping activity (e.g., number of traps) is not recorded and may vary over time, such data produce similar trend estimates for mammals as those from schemes that record effort, such as BBS (Noble et al. 2012). Data from 419 sites in England, Scotland and Wales that reported hedgehogs between 1981 and 2023 were included in the trend analyses, with an average of 83 contributing sites annually. Spatial density estimates, averaged over the second half of every decade, were obtained using data for the last four complete decades (i.e., to 2019).

### 2.2 Statistical Analyses

We estimated trends in non‐zero maximum counts and occurrence (presence–absence) data to separately identify changes in abundance and distribution, respectively. Analyses are summarised in Table 2. All analyses were conducted in Genstat (24th Edition, www.vsni.co.uk/genstat) and the code published on Zenodo (see Data Availability Statement).

**TABLE 2:** Summary of analyses.

Survey	Binomial trend	Poisson trend	Spatial analysis	Notes
Garden BirdWatch	Any presence	Maximum count excl. zeros	Probability of observation estimated at 20 × 20‐km grid cell level	Trends: a polynomial covariate term for week number (to allow for nonlinear, seasonal patterns). Spatial analysis: GLMM with binomial error structure; adjusted to account for year differences.
Living with Mammals	Any presence	Maximum count excl. zeros	n/a	Covariate term for survey effort.
Breeding Bird Survey	Any presence	n/a	n/a	
Mammals on Roads	n/a	Counts at 10 × 10‐km grid cell level	n/a	Logarithmic link function; covariate terms for recording method and logarithm of journey length.
National Gamebag Census	n/a	Standardised counts excl. zeros	Changes in relative abundance estimated for consecutive time periods	Trend: followed approach of Aebischer (2019); an offset (logarithm of site area) and covariate terms for site and year were included. Spatial analysis: contour bands shown representing smoothed hedgehog density.

#### 2.2.1 Analyses of Spatial Occurrence and Relative Abundance

Two datasets (GBW and NGC) were used to analyse spatial patterns of relative density:

- To estimate spatial occurrence, a subset of GBW data (weeks 20–35 inclusive, corresponding to the summer months and overlapping the survey period of LwM) was analysed. Annually, 15%–20% of sites recorded hedgehogs (from sightings or signs) in any 1 week. To estimate the probability of observing hedgehogs locally, a term for 20 × 20‐km OS grid cell was fitted in a generalised linear mixed model (GLMM) with year and week as covariates and grid cell as a random term; a binomial error distribution and logit link function were used. Scores predict the probability of observing hedgehogs on the logit scale, adjusting for differences due to year;
- Following the approach of Noble et al. (2012), changes in relative density between 1985 and 2019 were estimated using NGC data. Synoptic maps showing smoothed densities of hedgehog records were produced at a 10 × 10‐km grid cell resolution for two five‐year periods, 1985–89 and 2015–19, in order to be consistent with and comparable to maps produced by Noble et al. (2012) for 1995–99 and 2005–09.

The number of hedgehogs reported at each NGC site and year was standardised by dividing by site area before smoothing to give a density; sites of unknown area or with an area less than 1 km^2^ were excluded from the analysis. For each of the two new periods, a mean value for each site contributing data within the period was calculated and all values within a 35 km radius of the centre of each 10 × 10 km grid cell averaged to smooth the data. A radius value of 35 km provided an acceptable compromise between retaining spatial resolution and an adequate sample size of annual records (Noble et al. 2012). Data from 619 sites were included for the period 1985–89, and 788 sites, in the period 2015–2019. Smoothed data were categorised into six contour bands, using the same lower and upper limits as in Noble et al. (2012) for comparability. No information on numbers of tunnel traps or trap‐nights was available (only annual totals of animals caught or killed) because such information is not recorded in game books or part of an estate's annual shoot statistics.

Change maps corresponding to consecutive periods (e.g., 1985–89 and 1995–99) were produced in two steps: first, at each OS grid‐point, the smoothed value for 1985–89 was subtracted from the smoothed value for 1995–99; second, to determine contour levels of change, difference values were ranked by magnitude (i.e., ignoring the sign of the value) and the 10^th^, 40^th^ and 70^th^ percentiles calculated. This allowed a central band containing the 10% of values closest to 0 (denoted −10 to 10%), corresponding to no change, to be defined. Remaining values were split according to the 40^th^ and 70^th^ percentiles and cast into three bands if negative (corresponding to a decline), and three bands if positive (corresponding to an increase). These we denoted −100% to −70%, −70% to −40%, −40% to −10%, and 10% to 40%, 40% to 70% and 70% to 100% respectively. The same procedure was carried out for the pair of maps from the first and last decades, to highlight the spatial changes between the start and the end of the full 1981–2019 period.

#### 2.2.2 Trend and Change Estimates

##### 2.2.2.1 Changes in Numbers of NGC Records

National and regional estimates of change were calculated following Aebischer (2019). Non‐zero annual returns (i.e., those recording one or more hedgehogs) from sites with a minimum of 2 years' data were used in the analysis to estimate within‐site change. Counts were analysed using a generalised linear model (GLM) with a Poisson error distribution and logarithmic link function. Counts increased with site area and hence were standardised by using the logarithm of site area as an offset. Site and year were included as covariate terms.

Regional analyses were based on the former government office regions of England (ONS 2018), excluding London. With the exception of the East of England, regional analyses cover shorter periods than the full dataset because of insufficient site numbers in recent years. A minimum of three sites in any 1 year was used. Estimated coefficients were used to predict values for each combination of year and site on the logarithmic scale; these values were then averaged across all sites to give logarithmic annual indices. The index series was exponentiated to give annual indices on the arithmetic scale, and a generalised additive model (GAM) was fitted with one degree of freedom per decade or part‐decade. This degree of smoothing was based on a visual assessment of how well the fitted GAM smoothed out short‐term fluctuations while retaining the long‐term trend. GAMs were fitted to the longest available series and most were based on three to five degrees of freedom. Empirically, the choice did not constrain linear trends for very long periods but smoothed out fluctuations lasting one to 3 years while revealing the underlying longer‐term pattern of change.

The overall percentage change was estimated from the difference in smoothed values over the full data series (1981–2023) and over the last 10 and 25 years of the series (from 2013 and 1998, respectively). Ninety‐five per cent confidence intervals around annual index values were obtained by bootstrapping at the site level; confidence limits of percentage change values were obtained by fitting GAMs to each bootstrap sample, calculating the percentage change, and selecting the lower and upper 95^th^ percentiles of the 1000 values that resulted.

##### 2.2.2.2 BBS Trend

GAMs were fitted separately to each record type (namely, sightings of live animals, field signs, dead animals or local knowledge) and to all record types collectively. In logistic regression models, the relative frequency of local knowledge records was found to decline over time; however, models fitted to the data with and without the inclusion of local knowledge records showed similar trends.

A GAM with a binomial error structure and logit link function was fitted to the data and 95% confidence intervals obtained by bootstrapping at the site level. The baseline year is taken as 2002 to avoid issues with unrepresentative data collection in 2001 because of countryside access restrictions imposed by foot and mouth disease during that year.

Records of live hedgehogs were typically of a single animal, and little additional information could be gained from an analysis of counts as a quantitative variable.

##### 2.2.2.3 MoR Trend

For the analysis, journeys were restricted to those between 15 and 500 mi (24–805 km) to reduce records produced as short trial runs of the app or when the app was left running after active surveying had stopped.

It was not possible to identify the precise route of road journeys recorded on paper. Instead, 10 × 10 km grid cells were treated as sites and were included in the analysis if they contained start points, or any section of a journey, in two or more years. Where grid cells contained multiple journeys in a single year, all journeys were included. A model using covariate terms for recording method and logarithm of journey length was considered more robust than one using journey length as an offset because it was apparent that the relationship between casualty counts and journey length was not simple (see Section 4). Confidence intervals were obtained by bootstrapping at the site level.

##### 2.2.2.4 GBW and LwM Trends

Sites were included in analyses if they had taken part in at least two survey years; for LwM, a site was considered to have participated in a particular year if data had been submitted (including those weeks when no mammals were recorded) in at least 4 weeks.

Trends in LwM and GBW data were modelled in two ways: (i) to identify changes in the extent of spatial occurrence (i.e., the proportion of sites at different times where hedgehogs were present); and (ii) to identify changes in abundance between years at those sites for which maximum count data were available.

Trend analysis of GBW data used the same subset of data (weeks 20–35 inclusive) as that used for the spatial analysis. GAMs were fitted separately to presence‐absence and maximum count data, with binomial and Poisson error distributions respectively, for sites with data in two or more years and site‐week data for at least one mammal species. Cubic polynomials of week number were used as covariates to allow for nonlinear, seasonal patterns and model curves fitted with five degrees of freedom (Fewster et al. 2000), with 2009 as the baseline year.

GAMs fitted to LwM data included a covariate term for effort and a fixed effect to allow for all site differences. Site characteristics were not included explicitly as covariates in models because any effects are liable to be poorly estimated. Hence, for trend estimation, a cautionary approach was adopted.

Analyses of maximum counts only considered sites that recorded sightings in one or more weeks and averaged counts over those weeks for which effort was recorded.

#### 2.2.3 Changes in Site Characteristics Over Time

Information about site attributes was collected in only one survey, LwM, and included percentage coverage of vegetation or surface type (recorded as a category range, e.g., 26%–50%), and presence or absence of various features (Table 3). Estimates of percentage cover averaged over all sites in a year were calculated as the weighted mean of the mid‐point values of categories.

**TABLE 3:** Site attributes (vegetation/hard surface coverage and features) recorded in LwM.

Site attribute	Survey form value
Site type	Garden; Park/mown village green/residential square; Common land with rough grass or scrub; Wasteland/derelict land; Churchyard/cemetery; Playing field; Golf course; Allotment; Railway embankment/roadside verge; River bank/stream bank; Arable fields; Pasture; Woodland
Site established	Unknown; pre‐1900; 1900–1949; 1950–1999; after 2000
Site area	< 25 sq. m; 25–50 sq. m; 51–200 sq. m; > 200 sq. m
Percentage coverage: Grass; Shrubberies; Trees; Hard surfacing; Shed/building; Flowerbed	0; 1–25; 26–50; 51–75; 76–100
Percentage coverage left unattended	0; 1–25; 26–50; 51–75; 76–100
Features present on site	Garden shed; Pond/lake; Bat box; Hedgehog box; Bird box; Compost heap; Woodpile; Bird table; Bird bath; Bird feeder; Other animal feeder; River/stream; Fruit/berries; Nuts/seeds/cones; Bulbs/root vegetables; Leaf vegetables
Animals using site	Dogs; Domestic cats; Poultry; Grazing animals
Artificial illumination of site at night	Yes; No; Partial/intermittent
Boundary type: Open; Fenced/walled; Hedged (inc. trees)	None; Partially; Entirely
Boundary height	0–1 m; 1–2 m; > 2 m
Boundary holes/gaps	Yes; No
Boundary holes/gaps large enough to admit	Hedgehog; Fox; Roe deer
Features within 100 m of site	Gardens; Woodland; Common with rough grass or scrub; Pasture/grass field; Arable fields; Wasteland/derelict land; Park/mown village green/residential square; Pond/lake; River/stream; Churchyard/cemetery

To assess the change over time in percentage cover and frequency of features, GAMs or logistic regression curves respectively were fitted, using the same criteria as those for population trends, with one to four degrees of freedom and that with the lowest Akaike information criterion was selected. In addition, the same models were fitted using only sites in their first data year to assess whether such changes occurred more widely than only in those sites that participated in the survey repeatedly. The two groups of models were compared to determine whether changes in the characteristics of survey sites were representative of those occurring in the wider population of sites from which survey sites might be recruited. Because of the number of tests performed, *p*‐values for the significance of each variable were adjusted using the Benjamini–Hochberg procedure (Pike 2011) in order to control the false discovery rate within each group of variables.

#### 2.2.4 Urban and Rural Coverage

To assess the extent to which surveys were biassed in their coverage to either urban or rural sites, observations (i.e., site‐year combinations) of hedgehogs in GBW, LwM and BBS were assigned the 2011 rural/urban classification of the Office of National Statistics (ONS) output area in which each site was located (ONS 2025). ONS data were only available for England and Wales. Sites were located at the centroid identified by a postcode in the case of LwM, or at the midpoint of a 1 km^2^ grid cell identified by a grid reference in the case of GBW. The latter may overestimate the frequency of some rural categories due to instances where the site is in a built‐up area to one side of the grid cell, but the midpoint is classified as rural. The location of BBS sites was identified by the midpoint of a 1 km^2^ grid cell identified by the grid reference of the survey transect start point.

## 3 Results

### 3.1 Spatial Occurrence of Hedgehogs in Great Britain

The spatial pattern of occurrence of GBW hedgehog records (2007–2023) at a 20 × 20‐km grid cell resolution is shown in Figure 1. The predicted probability of observing one or more hedgehogs (*p*) was calculated as logit(*p*) = log(*p*/(1—*p*)), adjusting for differences due to year, and back‐transformed.

**FIGURE 1:**
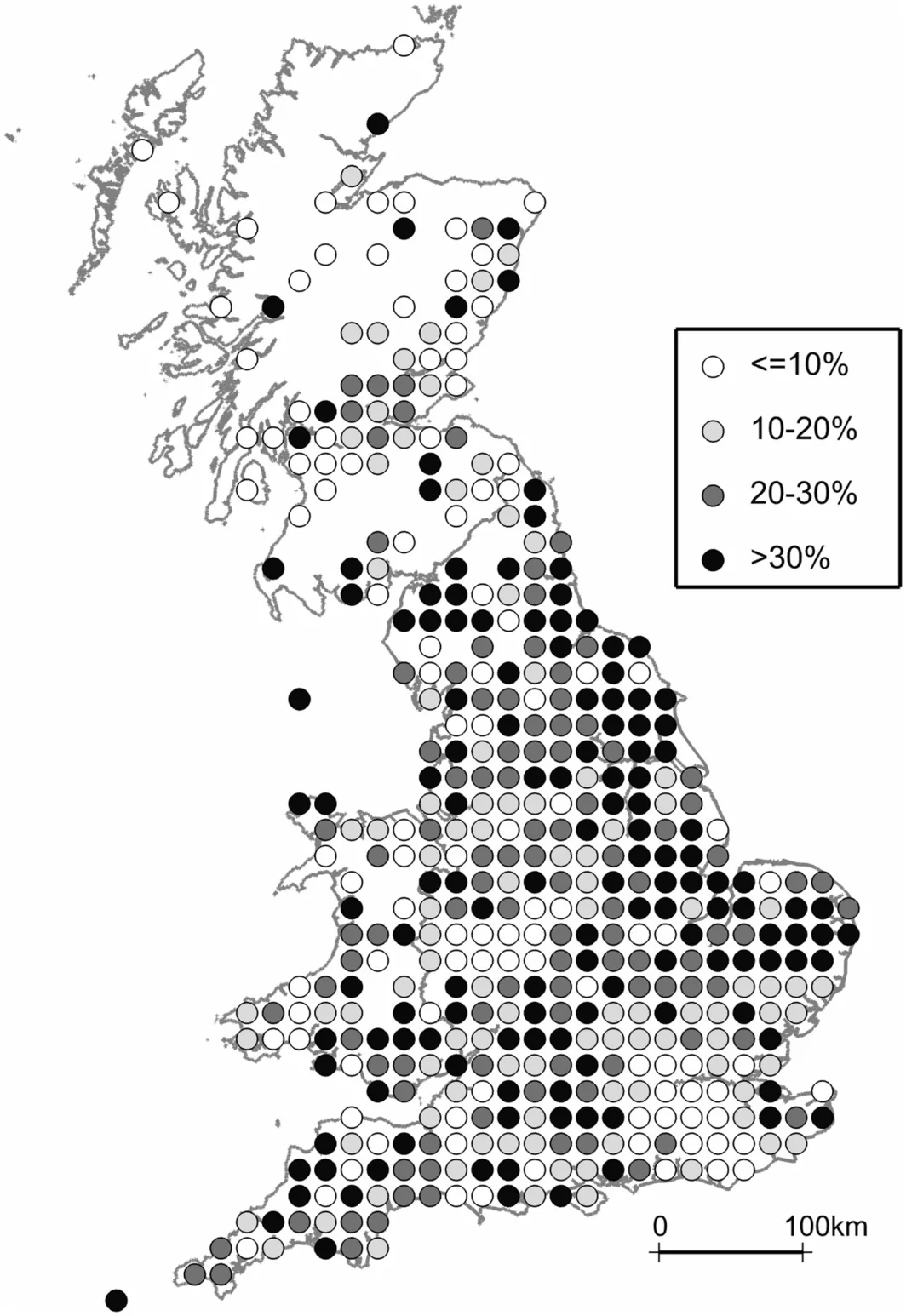
Hedgehog occurrence in England, Scotland and Wales expressed as the percentage probability that hedgehogs were observed in the *Garden BirdWatch*. Values were obtained by back‐transforming logit values. Darkest symbols indicate the highest observation rate.

### 3.2 Changes in Relative Abundance

Relative spatial abundances (estimated from NGC records) are shown on a coloured scale in Figure 2. Standardised counts were categorised into contour bands corresponding to 0%, 0%–20%, 20%–40%, 40%–60%, 60%–80% and 80%–100% percentiles.

**FIGURE 2:**
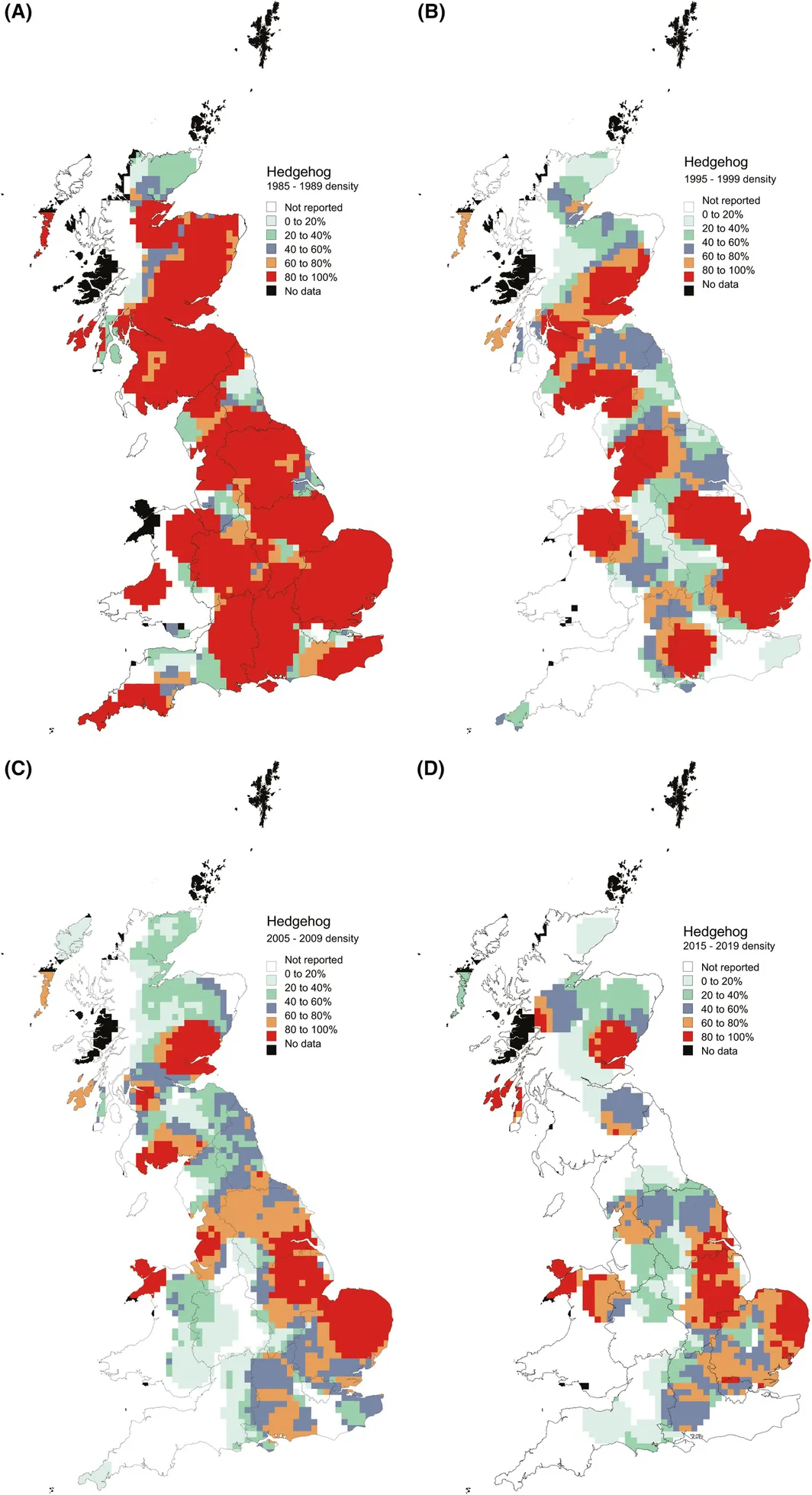
Maps showing the relative density of hedgehogs reported in the *National Gamebag Census* between 1985 and 2019 in four five‐year periods (A: 1985–89; B: 1995–99; C: 2005–09; and D: 2015–19). Annual numbers of hedgehogs at each site and year were divided by site area and averaged over the five‐year period. An average value for each 10 × 10‐km grid cell was calculated and is shown as a relative abundance. Pale blue indicates the lowest abundance and dark red, the highest. White areas are those in which no hedgehogs were reported.

Relative spatial densities estimated from counts submitted to the NGC in the 1980s suggest hedgehogs were widespread and comparatively abundant at this time. High‐density areas have become increasingly localised to eastern areas in subsequent decades, and by the 2010s are restricted to south‐east Scotland, north Wales and eastern England. Areas with no records of hedgehogs expanded eastward from the west coast over the same period and hedgehogs are no longer reported from most of western and northern Scotland (except on some Hebridean islands) and parts of the West Midlands, south‐east and south‐west regions and northern England by the 2010s. Over all four decades, declines in reporting are apparent across almost the whole of Great Britain.

### 3.3 National and Regional Estimates of Change

#### 3.3.1 NGC

The percentage of NGC sites that reported hedgehogs decreased from an average of 19.3% in the first 5 years (1981–1985; average 163 sites/year) to 2.7% in the last 5 years (2019–2023; 14 sites/year), with the largest change apparent in England (124 to 7 sites/year). The smoothed index for Great Britain, and for England and Scotland separately, decreased by more than 60% (Table 4). Average annual rates of change in England and Scotland were greater in more recent periods; for all NGC sites collectively, the mean rate over the last 10 and 25 years of the trend was −6.81 and −3.33% respectively, compared with −2.88% over the trend as a whole (1981–2023).

**TABLE 4:** National and regional percentage changes (with 95% confidence limits) in smoothed estimates of NGC hedgehog index over three time periods. Number of participating sites and start/end years of the period of data collection are also shown.

Country/region	Sites (*n*)	Start year	End year	Data period					
				From 1981		From 1998		From 2013	
				% change	95% CI	% change	95% CI	% change	95% CI
Great Britain	419	1981	2023	−70.7*	−83.3 to −49.7	−57.1*	−73.5 to −31.9	−50.6*	−70.4 to −13.9
England	288	1981	2023	−80.4*	−89.5 to −58.8	−70.0*	−81.6 to −40.0	−67.7	−79.3 to −25.9
Scotland	123	1981	2023	−64.9*	−87.6 to −23.7	−52.8*	−84.0 to −3.5	−35.3*	−81.1 to −6.5
Wales	8	Insufficient data							
South West England	20	1981	1993	−77.2*	−89.1 to −50.1	n/a		n/a	
South East England	48	1981	2017	−78.9*	−98.9 to −42.7	−3.4	−94.5 to 337.9	−12.7	−74.8 to 0.4
East of England	89	1981	2023	−83.7*	−92.8 to −60.2	−80.5*	−88.9 to −64.6	−80.6	−87.0 to −43.3
East Midlands	27	1981	2018	−70.1*	−86.5 to −38.2	−70.9*	−79.3 to −38.2	−0.8	−15.8 to 85.1
West Midlands	19	1981	1990	−84.0*	−99.9 to −39.3	n/a		n/a	
Yorkshire & Humber	46	1981	2019	−14.4	−48.2 to 51.6	24.9	−10.3 to 93.2	3.5	−11.7 to 25.2
North West England	19	1981	2009	−66.8	−94.4 to 62.0	−81.2	−97.2 to 1.0	n/a	
North East England	14	Insufficient data							

*Note:* Ninety‐five per cent confidence limits were obtained by bootstrapping.

* *p* < 0.05.

Insufficient sample sizes precluded calculation of trends in either Wales or the North East region of England for any of the time periods considered. Only the East of England had sufficient data to estimate a trend for the full period (1981–2023). For the other regions, too few estates reported hedgehog in recent years to calculate indices for the full time period. The confidence interval for the estimated change for the South East region of England (1998–2017) is notably large (−94.5% to 337.9%) and is a consequence of several factors: the change over this period is small, with the largest decline occurring between 1981 and 1998; because of this decline, the number of sites providing data after 1998 was small; and site‐level trends were highly variable from 2009 to 2014.

The largest regional declines, over varying time periods, are seen in the East of England, West Midlands, South East and South West regions (Table 4).

#### 3.3.2 NGC and MoR Trends

Smoothed trends show declines of 54.4% (2002–2013) and 70.7% (1981–2023) for MoR and NGC respectively (Figure 3).

**FIGURE 3:**
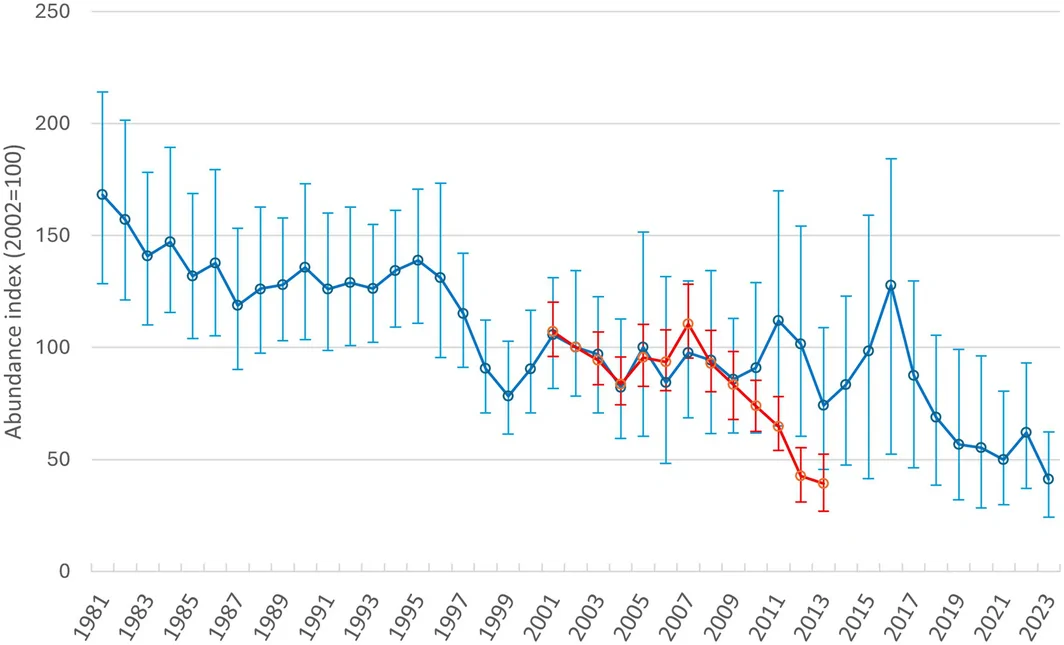
Estimated population change (indices of abundance) from NGC (1981–2023, blue) and MoR (2001–2013, red). Circles represent unsmoothed annual estimates with 95% confidence limits shown.

MoR records were categorised (Table 5) to investigate recording characteristics. Journey length is highly significant (*F* = 15.33 with 4 and 13,543 degrees of freedom, *p* < 0.001) in a model of logarithm of encounter rate.

**TABLE 5:** MoR records categorised by length, showing number of records (*N*), the proportion of journeys recording hedgehog casualties (‘positive journeys’), mean number of hedgehog casualties per journey and per 100 km.

Journey length (km)	*N* (journeys)	Proportion of positive journeys	Mean count/journey	Casualties/100 km
20–50	5741	0.3923	0.717	1.848
50–100	5253	0.4807	1.027	1.494
100–200	1837	0.5536	1.359	1.033
> 200	518	0.5598	1.672	0.615

Paper records had a significantly higher encounter rate than did other recording methods combined (*F* = 6.11 with 1 and 13,543 degrees of freedom, *p* = 0.013), which might be accounted for by the exclusion of some faster roads (namely, motorways and dual carriageways) in the original survey protocol but were included in other recording protocols. There is no interaction between this effect and that of journey length (*F* = 0.44 with 4 and 13,543 degrees of freedom, *p* = 0.778), suggesting the effects act independently of each other.

#### 3.3.3 BBS Trend

Individually and collectively, record types show strong negative trends over time. Figure 4 shows the trend for presence determined from all record types. The smoothed trend shows a decline of 80.4% (2002–2023).

**FIGURE 4:**
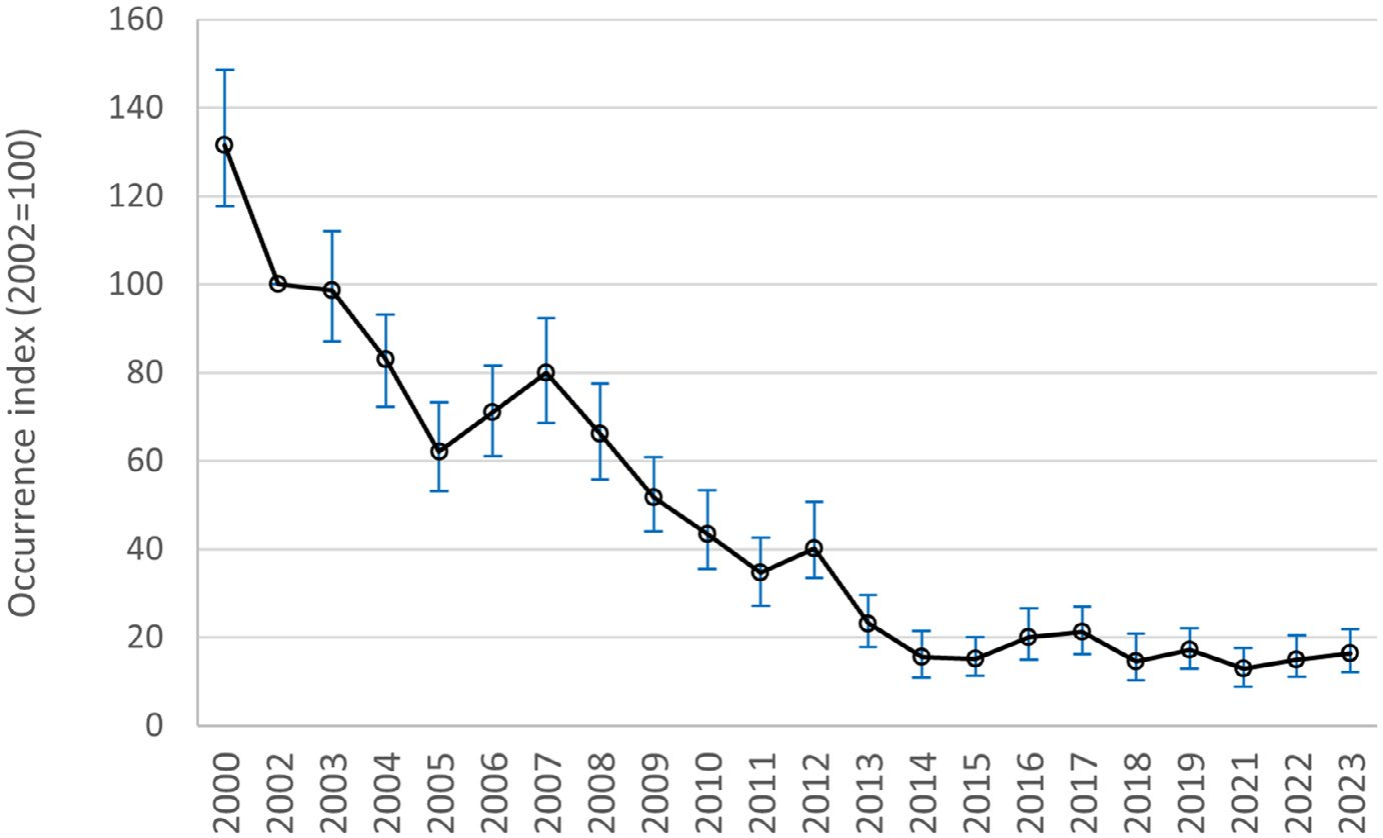
Population change (indices of occurrence) estimated from BBS (2000–2023). Circles represent unsmoothed annual estimates with 95% confidence limits shown.

#### 3.3.4 GBW and LwM Trends

GBW and LwM surveys recorded presence‐absence, from either sightings or signs, or maximum counts of mammals at sites in the built environment (predominantly domestic gardens in urban areas).

Smoothed population trend estimates from GAMs fitted separately to presence‐absence and count data from each survey are shown in Figures 5 and 6.

**FIGURE 5:**
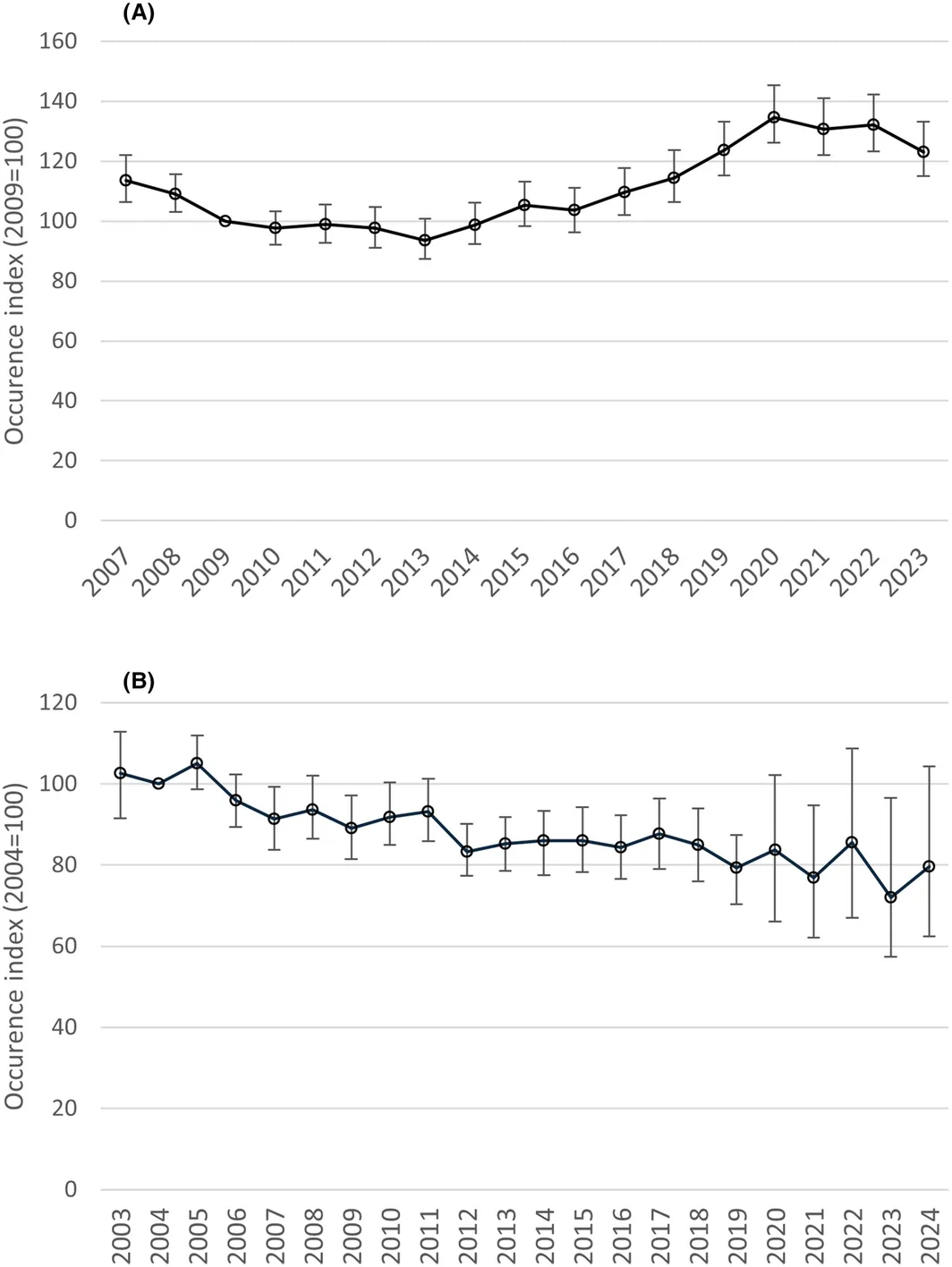
Proportion of sites recording hedgehog occurrence in: (A) GBW (2007–2023); and (B) LwM (2003–2024). Circles represent unsmoothed annual estimates with 95% confidence limits shown. Confidence intervals for LwM trend estimates relative to the baseline value increase in more recent years due to smaller sample sizes and to site ‘turnover’ such that later years share fewer sites with the baseline year than do earlier years.

**FIGURE 6:**
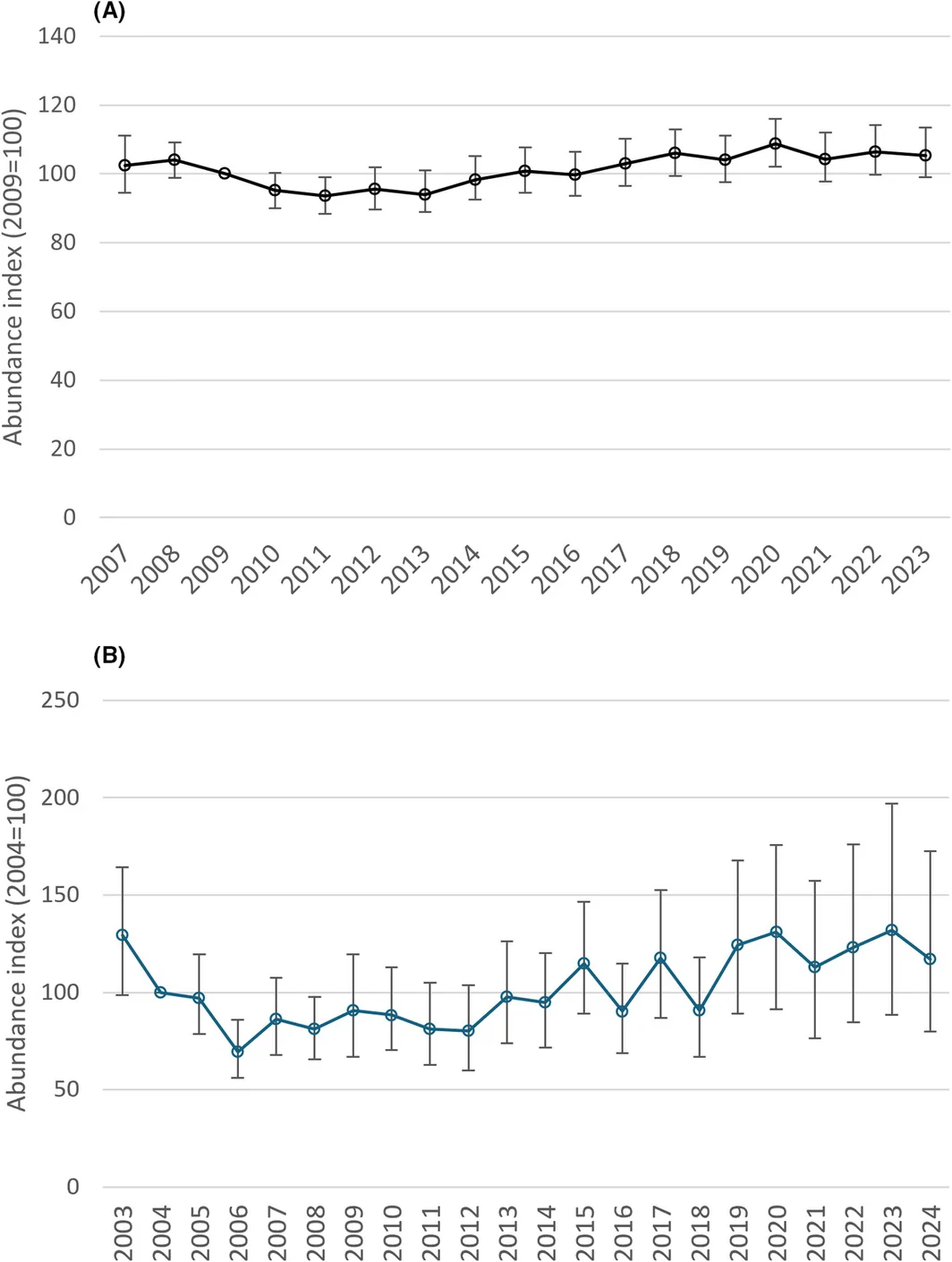
Average weekly maximum count, excluding zeros, at mostly urban sites as part of: (A) GBW; and (B) LwM. Circles represent unsmoothed annual estimates with 95% confidence limits shown.

Both GBW and LwM show an initial decline in the estimated proportion of sites recording hedgehogs. In the case of GBW (Figure 5A), the index increases to above the baseline value in 2017; in that of LwM (Figure 5B), the trend plateaus from 2012 to 2019, but with large variance in estimates from 2020 onwards. The overall change in the trend smoothed estimate in GBW, between 2009 (the baseline year) and 2023, is 24.0%, and in LwM, between 2004 and 2024, is −23.9% but with low precision.

To explore changes in abundance, GAMs were produced using non‐zero counts, that is, excluding site‐weeks when hedgehogs were not recorded. These are broadly similar for the two surveys (Figure 6A,B), initially decreasing and then increasing to above the baseline value (by 6.7% and 20.0% in their last year for GBW and LwM, respectively).

### 3.4 Site Characteristics and Features

Changes in the percentage cover of vegetation or surface type at sites in LwM between 2004 and 2024 are significant (*p* < 0.05) for all categories, with the exception of shrub coverage (Table 6a), although in some cases the overall direction of change is unclear. The absolute size of changes is small. Changes in the frequency of features are also significant (Table 6b) and typically larger (substantially so in some cases) than those in percentage cover. These changes are significant for sites in their first survey year, and for sites participating in two or more years, suggesting that changes in site characteristics are typical of sites more widely than only those that participate for a longer period (i.e., the effect of within‐site change is small).

**TABLE 6:** Changes in LwM site characteristics over time. Test statistics for GAM curves of percentage cover (a) and of the proportion of sites with a particular feature (b).

(a)					
Site cover	% change	df_1_	df_2_	*F*	*p* (adjusted)
Grass	−2.50	4	17	7.83	0.002
Shrub	1.04	1	20	4.13	0.056
Tree	−0.97	4	17	6.05	0.006
Hard surface	2.14	4	17	5.01	0.010
Building	1.31	1	20	14.89	0.002
Flower beds	1.87	4	16	12.97	< 0.001
Untended	1.56	4	17	4.57	0.013

(b)				
Site feature	% change	df	*Χ* ^2^	*p* (adjusted)
Shed	−4.78	3	18.29	< 0.001
Pond	8.25	1	13.60	< 0.001
Bat box	7.90	3	22.26	< 0.001
Hedgehog box	40.7	3	279.94	< 0.001
Bird box	7.69	3	32.99	< 0.001
Compost heap	−13.2	3	25.57	< 0.001
Wood pile	−5.93	3	47.77	< 0.001
Bird table	−7.86	2	13.06	0.001
Bird bath	4.99	4	57.83	< 0.001
Bird feeder	2.45	3	41.95	< 0.001
Other feeder	27.2	4	77.51	< 0.001
Watercourse	0.42	4	89.13	< 0.001

*Note:* Absolute percentage change is for the smoothed trend from 2004 to 2024.

### 3.5 Urban and Rural Coverage

Figures 7 and 8 show the proportion of site‐year combinations that recorded any mammal species (equivalent to the proportion of sites weighted by the number of years each site was surveyed) for each ONS category separately and for the six rural and four urban categories collectively.

**FIGURE 7:**
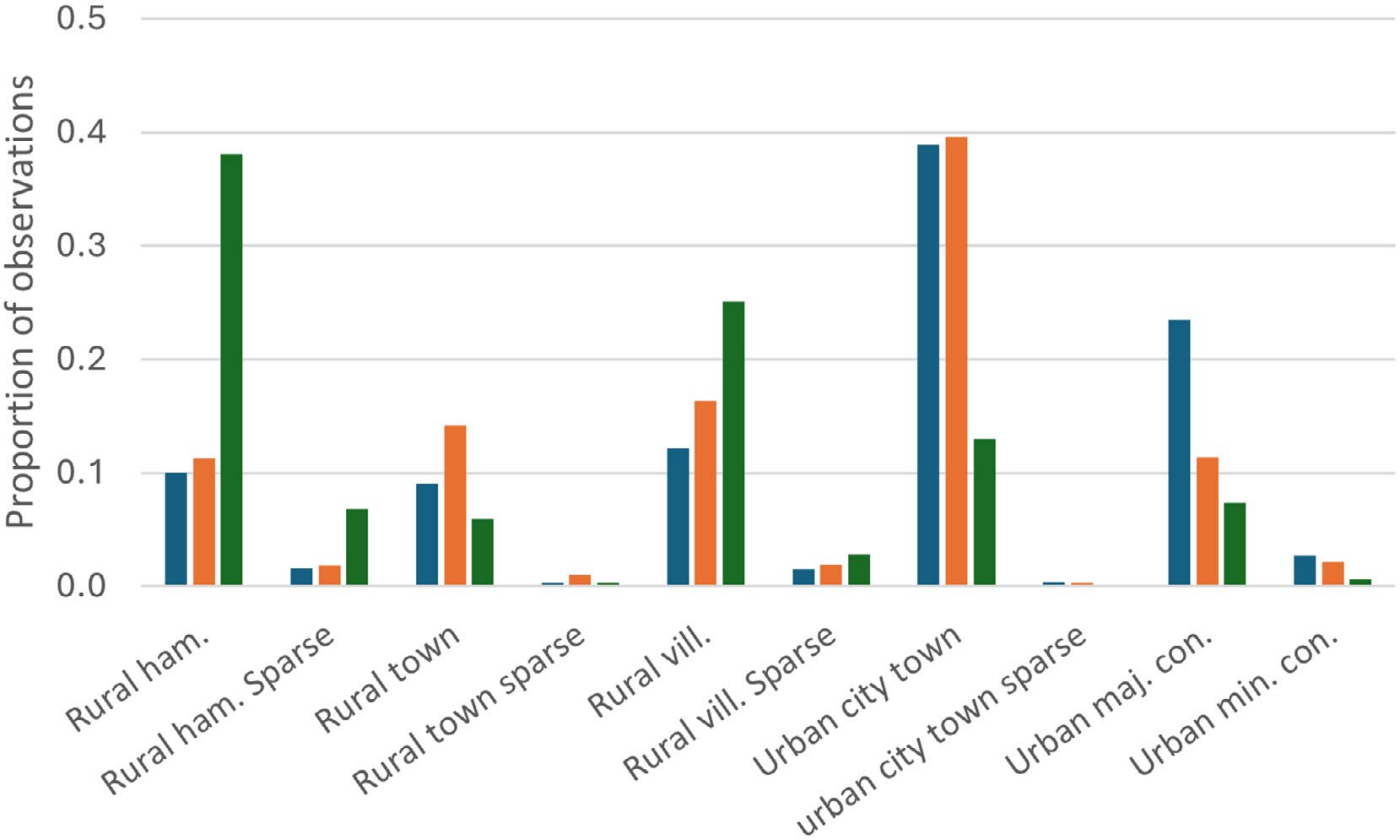
The proportion of observations in each ONS category for LwM (blue bars), GBW (orange) and BBS (green) sites in England and Wales. Categories: Rural hamlets and isolated dwellings (Rural ham.); rural hamlets and isolated dwellings in a sparse setting (Rural ham. sparse); rural town and fringe (Rural town); rural town and fringe in a sparse setting (Rural town sparse); rural village (Rural vill.); rural village in a sparse setting (Rural vill. sparse); urban city and town (Urban city town); urban city and town in a sparse setting (Urban city town sparse); urban major conurbation (Urban maj. con.); urban minor conurbation (Urban min. con.).

**FIGURE 8:**
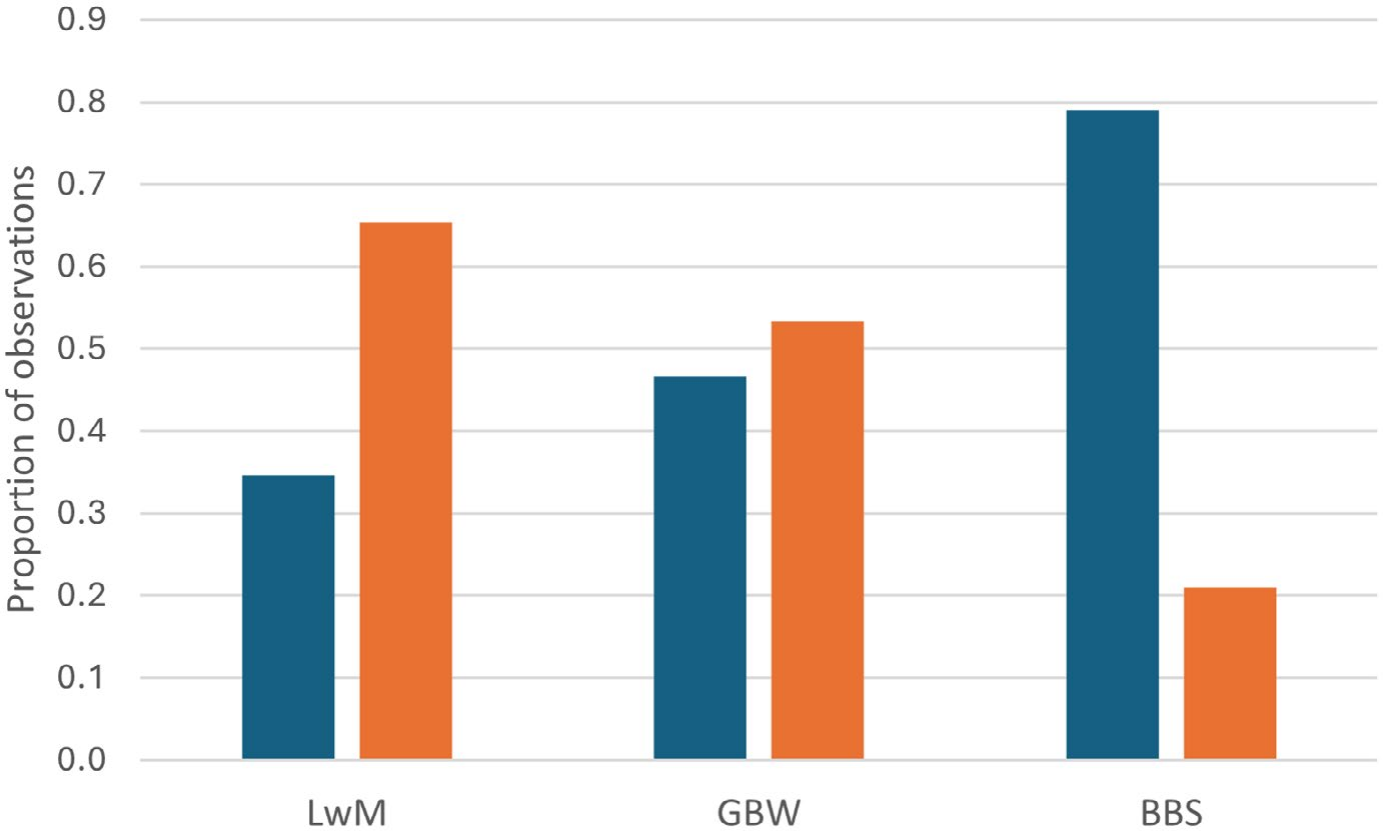
The proportion of observations in ‘rural’ (blue bars) and ‘urban’ (orange bars) categories in LwM, GBW and BBS.

## 4 Discussion

The five surveys analysed here, covering various periods between 1981 and 2024, suggest significant changes in the hedgehog population of England, Scotland and Wales over this time (Table 7), with substantial declines and apparent local extirpations across large parts of the country. Data from BBS, NGC and MoR show a longer‐term, widespread decline that has not recovered (Figures 3 and 4), while GBW and LwM show a smaller initial decline followed by a stable or even recovering urban hedgehog population more recently (Figures 5 and 6).

**TABLE 7:** A summary of the percentage change in population indices estimated over varying time periods for five surveys recording hedgehogs in Great Britain.

Survey	Index	Coverage	Period	% Change	95% confidence limits
NGC	Abundance	Rural	1981–2023	−70.7	−83.3 to −49.7
			1998–2023	−57.1	−73.5 to −31.9
MoR	Abundance	Rural	2002–2013	−54.4	−60.4 to −46.0
BBS	Occurrence	Rural	2002–2023	−80.4	−84.7 to −75.1
GBW	Abundance	Urban	2009–2023	6.67	1.4–12.7
	Occurrence			24.0	16.6 to 32.3
LwM	Abundance	Urban	2004–2024	20.0	−18.1 to 83.8
	Occurrence			−23.9	−41.3 to −1.72

Despite considerable uncertainty around estimates in some cases, similar trends are apparent in abundance and occurrence data, both between and within surveys, although representing very different methodologies. Perhaps the most striking feature of the data collectively is the apparent difference in trends from predominantly rural or urban sites, indicating an ongoing deterioration of the status of hedgehogs in rural areas. GBW and LwM focus on domestic sites, predominantly gardens, typically in towns and villages, that tend to support higher occupancy and densities of hedgehogs than those in the wider landscape (Williams et al. 2018; Hubert et al. 2011; van de Poel et al. 2015; Pettett et al. 2017; Schaus et al. 2020; Hitchcock et al. 2025). Moreover, hedgehogs will selectively use such environments in an agricultural landscape (Pettett et al. 2017). Changes in urban populations, however, are less clear, as contradictory trends are apparent (within and between metrics). Changes or differences in survey methodologies that might account for these are discussed below.

LwM and GBW sites are predominantly in ONS‐defined urban areas (Figure 8), although they span an urban–rural gradient across the built environment (Figure 7). Their distribution is less uniform than that of BBS, NGC and MoR records, which are predominantly in rural areas. Differences in population change apparent across urban and rural sites highlight the potential importance of conservation efforts in the built environment for this species. The importance of urban areas more widely in wildlife conservation is increasingly acknowledged (Ricketts and Imhoff 2003; Ives et al. 2016; Soanes and Lentini 2019), including that of some mammals (Boakes et al. 2024; Leivesley et al. 2021; Hof and Bright 2009; Lewanzik et al. 2022). The mechanisms by which such areas function to support wild species are incompletely understood (Lepczyk et al. 2017), but for various taxa they may act as refugia (Gentili et al. 2024; Vez‐Garzón et al. 2025) or provide nesting sites or protection from predators (Jokimäki et al. 2018). In the case of hedgehogs, this might also come about from differential habitat use and lower density of badgers ( *Meles meles* ), an intraguild predator of hedgehogs, in urban, compared with rural, areas (Lee et al. 2025), but may also be strongly influenced by the provision of food in residential gardens (Benjamin et al. 2025; Hitchcock et al. 2025).

Relative densities of hedgehogs nationally (estimated from NGC counts) decreased by 70.7% between 1981 and 2023 and show a progressive decline from west to east over time, with the largest declines in eastern England. If such a pattern was due to changing attitudes to reporting, it might be expected that increasing negative public attention on gamekeepers in upland areas managed for grouse shooting (Denny et al. 2021) would result in widespread non‐reporting of hedgehogs across the Pennines and Scottish uplands rather than starting in the south‐west and moving east. This lack of coincidence suggests that deliberate false non‐reporting is generally of reduced importance, and that hedgehog counts are genuinely lower over time. Recent occurrence remains highest in eastern and northern areas (Figures 1 and 2D), consistent with the analysis by Hof (2009) of opportunistic records submitted by members of the public between 2005 and 2007.

NGC and MoR produce abundance trends both showing declines in the period that they overlap (2002–2013) (Figure 3) and comparable to the decline in occurrence shown by the BBS trend. Together, these suggest a marked population reduction, in contrast to the trends from GBW and LwM. A possible explanation for the declines is a decrease in survey effort over time, for example, if survey participants become less motivated to look for hedgehogs because of a low encounter rate. To investigate this, we tried an occupancy modelling approach with BBS data, which is more robust to changes in effort. However, annual estimates from this were very variable. Trapping effort in NGC is thought to be broadly consistent and such data are comparable with estimates for other mammal species in the UK (Noble et al. 2012). Hedgehog records are a result of by‐catch taken in tunnel traps set for rats, stoats or weasels. A comparison of the proportion of sites in the first five years (1981–1985) and the last five years (2019–2023) shows capture rates for stoats and weasels to be similar in the two periods (36.3% vs. 37.6% and 33.3% vs. 31.1%, respectively; N.J. Aebischer, pers. obs.). This implies that trapping effort has not decreased, and that the substantial decline in the percentage of NGC sites reporting hedgehogs (from 19.3% in first five years to 2.7% in last five years, cf. Results) is real and specific to hedgehogs.

Extensive declines in previously common European species are increasingly described, including those of farmland bird and invertebrate species (Newton 2004; Mancini et al. 2023), common toad ( *Bufo bufo* ) (Petrovan et al. 2025) and mammals (Newson and Noble 2006). The large‐scale loss of common species is likely to impact ecosystem functioning, more so than the extinction of rare or threatened ones (Wagner 2020), and that of a widespread, generalist species such as hedgehog may be indicative of a wider ecological degradation of the landscape. Possible causes of the decline in rural hedgehog populations apparent in NGC, BBS and MoR are likely to be complex: loss of habitat diversity (Hof and Bright 2010a; Hof 2009) and reductions in prey availability (Hof and Bright 2010b) may be compounded by the effect of road mortality (Moore et al. 2020) and the interaction with, and predation from, badgers (Hof et al. 2019; Williams et al. 2018), which have likely undergone a substantial increase in population size since the 1980s (Sainsbury et al. 2019). The effects of badgers and road mortality on hedgehog abundance, however, may predominantly be at a local rather than landscape scale (Doncaster 1994; Moore et al. 2026). The underlying causes of the decline will have implications for conservation of species more broadly in agricultural landscapes.

### 4.1 Limitations of the Data

None of the surveys considered here were designed specifically to monitor hedgehogs and thus have various limitations, for example, a low detection rate of a nocturnal species monitored during the day, which has been highlighted previously as an inherent constraint for volunteer‐based surveys, primarily as a function of substantially lower detectability (Petrovan et al. 2011). We do not develop occupancy models for the occurrence data here and the observed changes may be confounded by detection probability (e.g., if habitat characteristics change or surveyor experience increases). Numbers of BBS surveys recording mammals, or indicating an intention to do so, are consistent, or increase, over the period of continuous decline in the BBS trend (2007–14), suggesting that, while an interest in mammal recording has been maintained across survey participants, participant behaviour (e.g., the level of search effort) may have decreased over time, perhaps because of the low frequency of mammal observations. Analyses might be improved by the use of multi‐species occupancy models (Royle and Dorazio 2006), which integrate data across species and sites, using coupled probability distributions, but these would constitute significant additional work beyond the capacity of the present study.

Occupancy models could be used to investigate LwM data further but given that site‐level detection probability may reflect local abundance, models would necessarily be restricted to the period between emergence of adults from hibernation and of offspring from the natal nest.

Hedgehog numbers reported to the NGC are a function of several factors: abundance of live animals; trapping effort; the susceptibility of hedgehogs to be captured; and the willingness of gamekeepers to report bycatch. The observed decline in hedgehogs therefore indicates a genuine decline in numbers of hedgehogs, and hence the number of sites reporting hedgehogs, but it may also reflect other factors, such as changing trap design and use of excluders, increased legislation to minimise the risk to non‐target species, and greater awareness of conservation issues among gamekeepers. It is unlikely, however, that so steep a decline is attributable solely to these factors.

Garden sites in LwM and GBW are unlikely to be representative of gardens more generally, given that survey participants self‐select and are likely to be motivated by an interest in wildlife, biasing any inference made from population trends. This is a common sampling bias in citizen science studies and is apparent in the high frequency of ‘wildlife‐friendly’ features reported at sites (e.g., approximately half of garden sites in LwM have hedgehog nest boxes in the most recent survey years). Increases in garden populations of hedgehogs may be the result of observed changes in percentage cover (of vegetation or surface type) and frequency of particular garden features (Tables 6a and 6b), which might be expected to correlate with increased hedgehog occupancy if they attract hedgehogs directly or correlate with other wildlife‐friendly actions. Moreover, trends may not be representative of those more widely if ongoing participation (and learning that hedgehogs are locally present) leads to additional efforts by participants to attract hedgehogs (e.g., with hedgehog nest boxes). No information was available about non‐participating sites, but it was possible to compare sites in their first survey year only (with the assumption that newly recruited sites are representative of the wider set of wildlife‐friendly sites) with sites that participated in two or more years (i.e., those used for trend estimates). Changes in sites characteristics were very similar in these two groups (the only discrepancy being in the increase of sites with bat boxes, which is unlikely, in and of itself, to have affected hedgehog occupancy). This suggests that, in this respect, the sites contributing to trend estimates are representative of at least those sites that might be motivated to participate. It remains the case, however, that these may be a minority of all garden sites.

Modelling LwM site covariates (e.g., in a mixed logistic regression model) might identify those characteristics that support hedgehogs in urban areas, assessing either the relationship between presence and site characteristics when first reported, or how presence changes in response to changes in site characteristics. Given the large number of sites and potential explanatory variables, data might be split into training and testing datasets to reduce the risks of spurious correlations (e.g., Langton et al. 2010). This might usefully form the basis of further study.

Abundance and occurrence indices for LwM and GBW describe similar population trends with the exception of that for LwM occurrence (Figure 5B). In contrast to the other trends, which show small or moderate increases, this plateaus below the baseline value. Species abundance and distribution tend to be linked such that changes in abundance are often positively correlated with changes in occupancy (Gaston et al. 2000). Measured abundance, however, is a function of true abundance and detectability, and an increase in the latter, from increasing trail camera use, for example, may explain the disparity between LwM abundance and occurrence trends. Some information about trail camera ownership by survey participants was available for LwM and indicated an increase in ownership over time. If this correlates positively with camera use in the survey, then it is likely to increase detectability and the likelihood of recording higher maximum counts. In this case, abundance might apparently increase while occupancy remains the same or decreases. Data identifying individual camera records, however, that could be used to investigate this further were not available for the majority of survey years.

A second possible explanation for the apparent differences between GBW and LwM trends is the way in which null records were identified in the two surveys. Participants in LwM were identified as having looked for hedgehogs in a particular week if there was an effort record for that week. In contrast, mammal recording is optional in GBW and there was no indication of whether a mammal survey had been carried out in a particular week other than that of a positive record. Null records for GBW, therefore (i.e., those surveys for which a species other than hedgehog had been recorded in one or more weeks and hedgehog sightings or signs had not been recorded) omitted those surveys in which participants had looked for mammals but not recorded any. As a result, the data might be biased towards positive records.

Changes in MoR methodology over the survey history (moving from paper‐based records to other forms of recording) are likely to be associated with different biases (August et al. 2020). It is also apparent that the relationship between counts and journey length is not simple: whilst both the proportion of journeys recording hedgehogs and the mean number of hedgehogs per journey increase with journey length, they do so nonlinearly, such that casualty rate decreases with journey length (Table 5). This might come about if longer journeys tend to be on faster roads and detection rate is lower at higher speeds and/or mortality rates are lower as fewer hedgehogs attempt to cross (and are killed on) wider, faster roads (Moore 2023).

## 5 Conclusion

Although the use of the data described here to identify changes in the hedgehog population is not without problems, and some trend estimates have a relatively high degree of uncertainty, these data collectively represent a huge amount of observer effort and are the best available over the past few decades. Moreover, trend estimates from separate national surveys and different metrics (counts and presence‐absence data) show similar, broadly consistent patterns in rural areas, supporting the directionality and extent of the apparent changes. Population trends in predominantly urban areas are not clearly defined by the data collected here, but do not show as marked a decline (in occurrence) as that in rural areas and show increases in abundance estimates. The difference in urban and rural trends is consistent with the positive correlation between hedgehog occupancy and the built environment, found by Williams et al. (2018). Although not analysed here, incorporating site characteristics as covariates might usefully be done in future work to identify features of urban environments that support hedgehog populations and the possible role of spatial niche partitioning alongside badgers (Lee et al. 2025).

There remains a clear need for a standardised hedgehog‐specific monitoring effort to target knowledge gaps, verify regional patterns and produce robust population density estimations in a variety of habitats. A current pilot study, utilising camera‐trap arrays in a citizen science framework, has the potential to address some of the limitations identified here (Cartledge et al. in prep).

## Author Contributions

**David E. Wembridge:** conceptualization (supporting), data curation (equal), formal analysis (supporting), project administration (equal), visualization (equal), writing – original draft (lead), writing – review and editing (equal). **Steve D. Langton:** formal analysis (equal), visualization (supporting), writing – review and editing (equal). **Henrietta Pringle:** writing – review and editing (equal). **Nicholas J. Aebischer:** formal analysis (equal), visualization (equal), writing – review and editing (equal). **Julie A. Ewald:** formal analysis (equal). **David G. Noble:** data curation (supporting), formal analysis (supporting), writing – review and editing (equal). **Silviu O. Petrovan:** supervision (equal), writing – review and editing (equal).

## Funding

Monitoring schemes were managed and funded by the Game and Wildlife Conservation Trust, the British Trust for Ornithology and the People's Trust for Endangered Species.

## Conflicts of Interest

The authors declare no conflicts of interest.

## Data Availability

BBS, GBW, LwM and MoR data supporting the findings of this study, and the Genstat code used for analyses, are published on Zenodo, at https://doi.org/10.5281/zenodo.20035490 and https://doi.org/10.5281/zenodo.21915729 respectively.
